# Should Emollients Be Recommended for the Prevention of Atopic Dermatitis?—New Evidence and Current State of Knowledge

**DOI:** 10.3390/jcm13030863

**Published:** 2024-02-01

**Authors:** Magdalena Grześk-Kaczyńska, Justyna Petrus-Halicka, Szymon Kaczyński, Zbigniew Bartuzi, Natalia Ukleja-Sokołowska

**Affiliations:** 1 Department and Clinic of Allergy, Clinical Immunology and Internal Diseases, Collegium Medicum in Bydgoszcz, Nicolaus Copernicus University in Torun, 87-100 Torun, Poland; justynapetrus@gmail.com (J.P.-H.); ukleja@10g.pl (N.U.-S.); 2 Department of Obstetrics, Gynecology and Gynecological Oncology, Faculty of Medicine, Collegium Medicum in Bydgoszcz, Nicolaus Copernicus University in Torun, 87-100 Torun, Poland; szkaczynski@gmail.com

**Keywords:** dermatitis, atopic, emollients, eczema, prevention and control, infant, newborn

## Abstract

Introduction: Atopic dermatitis (AD) is a chronic, pruritic skin disease with complex pathogenesis, which affects about 43 million children aged 1–4 years. One of the most known methods of alleviating symptoms of AD is emollient treatment, which varies depending on formulation and additional active ingredients. There is some evidence that emollients could be used in AD prevention in high-risk children. Materials and methods: A search of the literature from Cochrane Library, PubMed and Medline was conducted between August and September 2023 with the following keywords: “atopic dermatitis”, “emollients”, and “prevention”. Only randomised clinical trials published in the last 5 years were included into the meta-analysis. Results: Considering the inclusion criteria only 11 randomized clinical trials were taken into account, and six of them proved lack of effect of emollients in the prevention of atopic dermatitis among neonates from AD risk groups. Conclusions: Emollient treatment has a good safety profile and most of the ingredients used in formulations are nonirritant for sensitive newborn and infant skin. There is some evidence of the positive effects of emollient treatment in prevention of AD in predisposed populations. The relatively high cost of emollient treatment (vs regular infant skin-care routine) would support the necessity for further evaluation of their effectiveness in nonpredisposed populations.

## 1. Introduction

Atopic dermatitis (AD) is a chronic, pruritic skin disease with complex pathogenesis. Children affected by this condition first develop symptoms within 6 months, 1 year, and 5 years of age in 45%, 60%, and 89% of all cases, respectively [1]. Many environmental factors influence the development of AD, such as skin barrier dysfunction, cutaneous and systemic immune dysregulation, skin microbiota dysbiosis, and genetics [2]. According to the literature on AD, the frequency of the disease in the pediatric population can be as high as 20%, whereas among adults, it ranges from 7 to 14% [3]. The World Health Organization’s Global Report on Atopic Dermatitis states that the disease affects about 223 million people, and 43 million of these are children aged 1–4 years [4].

### 1.1. Pathogenesis of AD

Genetics play a major role in the development of AD. Genetic disorders vary between children and adults and affect the epidermal barrier, innate or adaptive immune response mechanisms, and interleukin genes (IL-25, TSLP [thymic stromal lymphoprotein], and IL-33), as well as genes involved in vitamin D metabolism and synthesis receptors [5]. The genetic and structural abnormalities observed in the epidermal barrier in AD patients negatively affect many essential skin functions and lead to symptoms. One mentioned abnormality is mutations in the filaggrin gene (FLG), which is a major risk factor in the development of AD, along with a family history of atopy [6]. Mutations in the FLG increase the AD risk by 3–5 times [7]. Null mutations are observed in about half the patients with moderate-to-severe AD. Heterozygous carriers have an estimated eight-fold risk of the disease, while carriers of two mutated alleles are virtually always affected (with an odds ratio of >150) [5].

The structure and immune response of the epidermis plays an important role in the development of atopic diseases, along with genetics. The epidermis acts as a physical barrier, reducing water loss and protecting tissues from external damage. Additionally, it impacts immune response mechanisms. The key elements in the pathomechanism of AD are the immunological processes in the skin. Elevated levels of IgE antibodies and eosinophilia are observed among most patients affected by AD. When the immune response itself is evaluated, a predominance of type 2 inflammation can be observed, characterized by the overproduction of interleukins, such as IL-4, IL-5, and IL-13. Excessive cytokine production is correlated with the disease’s severity [8]. Furthermore, influenced by mechanical trauma, infectious agents, and allergens, the inflammatory process is activated, which releases TSLP—a cytokine similar to IL-7, as well as IL-4, IL-13, IL-25, and IL-33 [9,10]. Keratinocytes are the primary cells forming the epidermis, and their autocrine function plays a crucial role in the skin’s immune response. They produce pro-inflammatory cytokines, notably IL-25, IL-33, and TSLP. The activation process of additional cells, such as dendritic cells and mast cells, triggered by TSLP, is particularly evident among patients affected by AD [11]. A vital group of pro-inflammatory compounds includes keratinocyte-stimulating kallikreins. Research has indicated that kallikreins 5 and 7 increase IL-4 and IL-13 production [12]. Another significant element of immune response is nitric oxide, which takes part in the activation of the gaseous mediator pathway [13,14]. Recent studies have shown that the skin T helper (Th) cells in infants, young children, and adolescents are polarized in the Th2 and Th17 axes, with the absence of the Th1 upregulation observed in adults with AD. Moreover, in young children, inflammation signs can be observed before the disease’s development, which indicates the need for AD prevention [1].

### 1.2. Skin Barrier in AD and Emollient Types

Skin barrier dysfunction is a significant indicator of AD development. Kelleher and colleagues determined that transepidermal water loss in the first months of life may be a predictive factor for AD development [15]. The treatment of AD is challenging, notably among children. Depending on the severity of the disease, it may be systemic or topical. Affected areas require regular administration of emollients, topical corticosteroids, and topical calcineurin inhibitors. Emollients are topical formulations with vehicle-type substances lacking active ingredients; they provide a physical barrier for the skin. Emollients typically contain petrolatum, paraffin, glycerine, and plant-derived butter and oils [16]. Their use in AD treatment can be explained by their ability to restore and maintain the impaired skin barrier. Zhang J et al. proved that the usage of emollient-containing linoleic acid-ceramide not only alleviates symptoms such as skin inflammation but also alters the immunological response by decreasing the TSLP and IgE levels [17]. Emollients containing ceramides have also been proven by Shindo S et al. to ameliorate the cutaneous barrier function and increase the ceramide levels in the stratum corneum [18].

The use of systemic drugs such as corticosteroids and off-label drugs (cyclosporine, methotrexate, or azathioprine) is complex due to their restrictions and potential side effects. Besides the substances described above, other treatments are also available for AD, such as phototherapy and biologic medication. There are also new safe drugs for AD treatment in children, such as dupilumab [1]. Phototherapy is a treatment option recommended particularly for adults but may also be used among children.

For years, emollients have played a fundamental role in preventing and alleviating AD symptoms. One of their main functions is protection against water loss (TEWL—transepidermal water loss), and depending on the type and additional ingredients, they have moisturizing, anti-inflammatory, and antipruritic properties. AD is a disease that impacts the affected individual’s quality of life and causes economic burdens. The complex pathogenesis of AD makes its treatment challenging. The basic treatment of AD requires rebuilding the skin barrier by hydrating and lubricating the skin. When the findings of the recent research papers on the preventive role of emollients in AD treatment were analyzed, the differences evident in the substances used and the method of application were worth noticing. Emollient products usually consist of humectants that hydrate and occludents that inhibit water evaporation. Such a combination of ingredients specifically helps patients suffering from AD [19].

**Figure 1:**
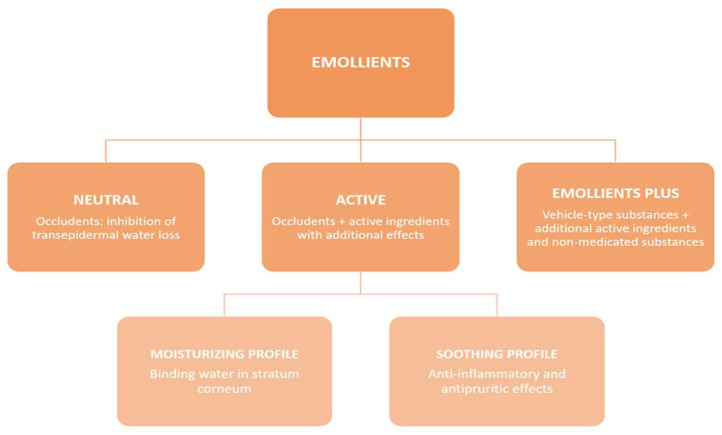
The basic division of emollients groups.

Clinically, emollients can be divided into two main groups: neutral and active. Neutral emollients consist only of an occludent that inhibits TEWL. The active group can be further branched out into emollients with a moisturizing profile and those with a soothing profile (such as an anti-inflammatory or antipruritic) (Figure 1).

The European guidelines on AD strongly recommend emollient application after a bath. Only products free of protein allergens or haptens, which can cause contact allergy (such as lanolin/wool wax alcohol or preservatives such as methylisothiazolinone) should be used, especially among children under 2 years old. Emollients should be applied daily in sufficient quantity, and patients should adjust the frequency of application as per degree of skin dryness [20].

The Polish Society of Allergology, Polish Society of Dermatology, Polish Society of Paediatrics, and Polish Society of Family Medicine (PTMR) published interdisciplinary diagnostic and therapeutic recommendations on AD, in which emollients were described as the basis of AD treatment and that they should be used two-to-three times per day, 250–500 g per week: minimum 200 g in children and 500 g in adults. The recommended emollients were devoid of allergens and haptens. According to the recommendations, primary prevention of AD is recommended with inter alia emollient use from the first day of life [21].

The Clinical Practice Guidelines for the Management of Atopic Dermatitis by the Japanese Dermatological Association suggest emollients as one of the three primary measures in AD treatment [22]. In another consensus guideline for the management of AD, an Asia-Pacific perspective is that emollients should be used two-to-three times per day or as frequently as the skin gets dry, depending on the climate and the use of air conditioners. The quantities of emollients used should be within 100–200 g per week for children and 200–300 g per week for adults; additional applications should also be considered before or after swimming and bathing within 5 min [23]. The European guidelines (EuroGui Derm) on AD recommend daily emollient application, immediately after bathing or showering. The recommended amount of emollient sufficient to achieve optimal effects oscillates around 250 g/week. Application of the product may follow the fingertip unit rule: “a fingertip unit (FTU) is the amount of ointment expressed from a tube with 5 mm diameter nozzle and measured from the distal skin crease to the tip of the index finger (ca. 0.5 g): this is adequate for application to two adult palm areas, which is approximately 2% of an adult body surface area“ [20]. It should be mentioned that the latest guidelines do not answer the question about the superiority of any specific type of emollient. Ridd et al. found no significant difference between the four emollient types (lotions, creams, gels, or ointments) in the context of alleviating symptoms of childhood eczema [24].

Elena Galli et al. distinguished three types of emollients by generation. Among the first generation were hygroscopic and occlusive emollients (vaseline, paraffin oil, fatty alcohols) and hydrophilic polymers (collagen, hyaluronic acid, chitosan, and polysaccharides gelling). The second generation included emollients that hydrate the skin and rebuild the skin barrier: glycerol, sorbitol, natural moisturizing factor substitutes, derivatives of pyrrolidone carboxylic acid, urea (5–10%), lactic acid, and ammonium lactate. The third generation included emollients used in barrier skin therapy, such as physiological lipids (ceramides, cholesterol, and polyunsaturated fatty acids) [25].

Furthermore, there are also products described as “emollients plus”, which are topical formulations with vehicle-type substances and active, nonmedicated substances such as saponins, flavonoids, e.g., licochalcone A, riboflavins from protein-free oat plantlet extracts, and bacterial lysates, e.g., Aquaphilus dolomiae, Vitreoscilla filiformis, or a synthetic derivative of menthol such as menthoxypropanediol [16]. Emollients also have an anti-inflammatory effect by cytokine inhibition (TSLP, IL-18, IL-2, IL-12, IL-17, IFN-γ, IL-1β, TNF-α, IL-4) and chemokines (MCP3/CCL7, MDC/CCL22, MIP-3α/CCL20) [26], as well as an antipruritic effect [27]. Additionally, they support congenital immunity by activating TLR2, TLR4, TLR5, and natural antibacterial peptides (hBD-2, cathelicidins LL-37, and psoriazines) [28]. Emollients also inhibit the growth of Staphylococcus aureus, but do not harm the skin microbiome [20,29,30].

### 1.3. Study Justification and the Aim

The question remains: should emollients be used during the first months of life to prevent AD in all children? Or should it be used only in children with risk factors of AD? Or, perhaps, this intervention does not influence the future condition of the skin and is an unnecessary expense and burden on the family.

This study aims to evaluate whether emollients are effective in preventing AD in high-risk infants.

## 2. Materials and Methods

**Figure 2:**
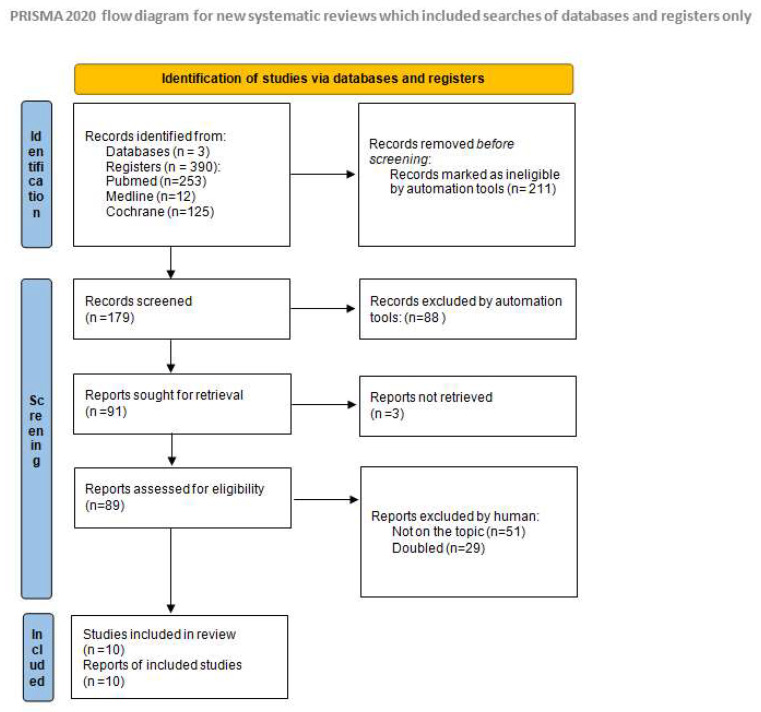
Identification of studies via databases and registers [31].

The systematic review and literature analysis was based on a search in PubMed base, Cochrane Library and Medline conducted in August–September 2023. In our research, we used the following keywords: “atopic dermatitis”, “emollients”, and “prevention”, and received 253 results in PubMed, 66 results in Cochrane Library, and 12 in Medline (Figure 2). Prevention was defined as a negative diagnosis of AD during the selected study follow-up.

The studies included in the analysis had to meet the following inclusion criteria:

- Randomized clinical trials or metanalyses;
- Neonates from the group of AD risk (parents’ history of atopy);
- Studies published in the last 5 years.

We excluded papers where the patients were above the neonatal age, nonrandomized studies, and studies with publication dates before 2018.

Registration statement: This review was not registered in PROSPERO.

## 3. Results

Based on the inclusion and exclusion criteria, we collected 10 studies published within the last 5 years (Table 1). The selected randomized clinical trials were analyzed, considering the most important aspects of characteristics among study groups, specific, intervention methodology, including type of emollient and methods of application, endpoints of intervention and results.

In the study by Ní Chaoimh et al. [26], AD is defined by the UK working party refinement of the Hanifin and Rajka diagnostic criteria, and AD risk is defined as a parental history of AD, asthma, or allergic rhinitis. The ceramide enriched emollient is used from birth until 2 months of age, and the conclusion from the study is that emollients effectively prevent AD.

In the study by Bradshaw LE et al. [32], the AD definition is identical to the one used in the research above, and AD risk is defined as a parental report of a clinical diagnosis of AD and food allergy. The BEEP trial (2023) [32] did not prove the protective role of emollients and included 1394 neonates, the follow-up period being the longest of all trials analyzed. Among the analyzed infants, emollient use exceeded thrice per week, and a single application covered the entire body. The study used basic petroleum emollients. The BEEP trial also assessed food allergy prevention by emollient use. Results for the control and intervention groups were the same for the food allergy risk at 5 years, and the results for AD diagnosis were comparable in both groups at 3, 4, and 5 years. The conclusion from the study was that emollients do not prevent AD development.

The definition of AD in a study by Skjerven HO et al. [33] corresponds to that in the studies above, and the AD risk is defined as parents with an atopy history. PreventADALL study gathered 2379 neonates, among whom 597 were in the emollients intervention group and 583 were assigned to the combined intervention group. The study explored whether the emollient application or early complementary feeding reduced the development of AD by the age of 12 months. Paraffin-based emollients were used in the study. Throughout the research, parents were asked to apply the emollient to the entire faces of their children included in the study after baths for 5–10 min with added emulsified oil at least 4 days per week, from the age of 2 weeks to 8 months. Follow-up lasted until 12 months of age.

In the study by Techasatian L et al. [34], the definition of AD is based on the Hanifin and Rajka diagnostic criteria, and the AD risk group is defined as having a parent who has (or had) physician-diagnosed AD, asthma, or allergic rhinitis. In the study, 154 participants had different emollients to choose from—four with additional substances and one petrolatum-based. The study was conducted in Thailand. The study concluded that emollients used for skin dryness protect infants against AD development in a tropical climate.

A study by McClanahan D et al. [15] defines AD as a continuous or intermittent itchy skin condition present for 1 month or more in addition to three of the following: a history of rash in the skin creases, generally dry skin, flexural dermatitis, first-degree relative with hay fever or asthma, and an onset younger than 2 years old. The AD risk group is defined as having a first-degree relative with a history of AD, asthma, or allergic rhinitis. Emollients used in the study are ceramide enriched emollients. The conclusion from the study is that emollients are ineffective in AD prevention.

Dissanayake E et al. [35] define AD as a case of an itchy skin condition in the last 12 months, and children in the study were not among the risk groups, although it was considered in the final results. The risk group was characterized as having a family history of allergic diseases. Emollients used in the study were with the content of cermides, cholesterol, and free fatty acids.

In the small-scale study with a participant group of 50 infants by Harder I et al. [36], the AD definition is a case of eczema occurring at any time of the intervention, and the AD risk is defined as ≥1 first-degree relative with physician-diagnosed asthma, AD, or allergic rhinitis. Emollients used in the study contained a prebiotic Vitreos-cilla filiformis lysate. The conclusion from the study is that the emollients do not prevent AD.

In the PEBBLES study, the AD definition is not provided, and the children from the AD risk groups are defined as having a family history of allergic disease [37]. In the study, ceramide-rich emollient was used twice a day to the full skin surface. The study supports the preventive role of emollients in AD prevention.

Bellemere G et al. [38,39] do not share the AD definition in their study: AD risk is de-fined as having two atopic parents. Information about the type of emollient is missing. Emollient was used twice a day, and cleansing cream and bath oil twice a week from the same brand. In the study, 120 newborns with atopy risk were included and divided into prevention and control groups. In parallel, 60 newborns with no atopy history were followed. A relative risk reduction of 54% due to emollient applications was observed.

Kottner J et al. [40] define AD with the Simpson criteria [41] as having at least one first-degree family member with physician-diagnosed AD, asthma, or allergic rhinitis/rhinoconjunctivitis. In the study [40], 160 infants with 52 weeks intervention and 52 weeks follow-up participated. The intervention was daily leave-on emollient application (lipid content 21%), the control group used normal skin-care routine. There was no statistical difference in AD incidence in both groups, but the AD severity was higher in the control group.

Studies differ in the way of the emollient application, the body surface of the emollient-lubricated skin, and the type of emollient. Control visits in presented studies vary in frequency, and some of them were held online [26,32,33,34,35,36,37].

**Table 1:** Summary of the most important clinical trials on the role of emollients in the prevention of AD published in the last 5 years. FA-food allergy.

Study Title and Type of Study	Participants	Method of Application	Emollient Type:	Results
Ní Chaoimh C et al. (2022) [26] Randomized controlled clinical trial	*N* = 321 infants 161 intervention and 160 control	Intervention group: Emollient use twice-daily, whole body except fingertip quantitation from scalp, implemented within days of life for the first 8 weeks Control group: standard routine skin-care advice	AVEENO^®^ Dermexa Fast & Long Lasting Balm (Johnson & Johnson Santé Beauté France, JJSBF)—formulation with added ceramides, oat ingredients, fatty acids.	Daily emollient use until 2 months of age reduces the incidence of AD in the first year of life in high-risk infants.
Harder I et al. (2023) [36] Randomized controlled trial	*N* = 50 neonates	Intervention group: skin-care advice plus emollient 1 per day for 1 year Control group: general infant skin-care	Emollient containing a prebiotic Vitreos-cilla filiformis lysate	Daily emollient use did not significantly reduce the risk of developing AD or impact skin physiology development
Bradshaw LE et al. (2023) [32] Randomized controlled trial	*N* = 1394 term infants 693 emollient group; 701 controls	Intervention group: Emollient all over the body daily for the first year, for >3 days per week plus standard skin-care advice control—standard skin-care advice only. Emollients implemented 11 days of life.	Basic petroleum emollients	Daily emollient application during the first year of life does not prevent atopic dermatitis.
Skjerven HO et al. (2020) [33] Cluster randomised trial	*N* = 2397 newborn infants Assigned to different intervention groups	Intervention group: baths for 5–10 min with added emulsified oil and emollient applied to the entire face after the bath on at least 4 days per week, from age of 2 weeks to 8 months	Paraffin-based formulations	Skin emollients did not reduce development of atopic dermatitis by age 12 months.
McClanahan D et al. (2019) [15] Randomised controlled trial	*N* = 100 newborn infants	Intervention group: daily to all body surfaces excluding the scalp and diaper area	Emollient with shea, pseudoceramide-5 and two FLG breakdown products—arginine and sodium pyrrolidone carboxylic acid	No statistically significant effect in atopic dermatitis prevention of the ceramide and amino acid-containing emollient;
Techasatian L et al. (2021) [34] Randomised controlled study	*N* = 154 neonates 77 intervention group 77 control group	Intervention group: Once daily to the baby’s entire body surface (excluding the scalp), starting as soon as possible after birth (within a maximum of 3 weeks) till 6 months of age.	5 types of emollients to choose: Four claimed to be therapeutic emollients, with a variety of anti-inflammatory ingredients. One is basic petrolatum-based emollient.	In tropical climate emollients put on skin in case of skin dryness protect infants against AD
Dissanayake E (2019) [35] Randomised Controlled study	*N* = 549 babies qualified to be randomized, 459 infants completed the intervention	Intervention group: 2–3 times/day, after a bath or on clean skin, particularly on the cheeks and the peri-oral area.	Cream containing ceramide, cholesterol, and free fatty acids	Emollient did not show any effect on reducing the development of AD and FA at 1 year of age
Lowe AJ et al. (2018) [37] Randomised trial	*N* = 80 children	Intervention group: Within the first three weeks 6 g of EpiCeram ™ to the full skin surface of their child twice per day	6 g of EpiCeram complex ceramide-rich emollients	twice daily prophylactic use of a ceramide dominant emollient, reduced incidence of AD
Bellemere, G et al. (2019) [38,39] Randomised controlled trial	*N*= 120 infants	Intervention group: balm twice a day, cleansing cream and bath oil twice a week Control group: Standard skin-care	No information given	The beneficial effect of prevention maintained after 24 months of follow-up.
Kottner J et al. (2022) [40] Randomised trial	*N* = 160 infants	Intervention group: skin-care regimen including once daily leave-on product application Control group: standard skin-care	Lipd content 21%	No effect in prevention

An overview of six recently published meta-analyses examining emollient usage to prevent AD in infants demonstrated that most of the literature shows no statistically significant difference in the incidence rate of AD among neonates subjected to emollient therapy and those with standard skin-care (Table 2). This conclusion did not apply to neonates with risk factors of AD development, among whom such an intervention decreases disease incidence. The meta-analyses emphasized the complications and negative aspects of emollient use, such as the increased risk of skin infectious diseases among neonates treated with emollients [42,43,44,45,46,47].

In the meta-analysis by Zhong Y et al. [46], a significant benefit of the usage of prophylactic emollients was observed among the high-risk population (RR 0.75, 95% CI 0.62–1.11). In the meta-analysis by Xu et al. [42], six of the nine studies included supported early emollient use, while three did not. The meta-analysis by Kelleher MM proved that skin-care interventions such as emollient application during the first year of life in healthy infants may prove ineffective in preventing eczema and increase the risk of food allergy and the risk of skin infections [44].

The meta-analysis by Youjia Zhong et al. [46] proved that reductions in AD risk depend on the study population’s risk, age at outcome assessment, and treatment duration. AD development was demonstrably reduced up to 6 months of age in all populations and was efficacious up to 12 months of age in high-risk populations. Furthermore, the protective effect was observed when the AD outcome was assessed, and the parents were still regularly treating their children with emollients.

## 4. Discussion

The studies included in this meta-analysis were evaluated to determine the effectiveness of the application of emollients in AD prevention. Considering the inclusion criteria, only 11 randomized clinical trials were considered; 5 proved that emollients are beneficial in AD prevention, and 6 demonstrated that emollients are ineffective in preventing AD among neonates from AD-risk groups.

Different types of emollients were used in the included publications. Products containing ceramides were applied in two of the studies that proved the protective role of emollients [26,37]. In their study, Techasatian L et al. selected various types of emollients, with four out of five containing additional substances [34], whereas in two of the remaining studies, the information considering the specifics of applied products was not provided. Studies that did not prove the beneficial effect of emollients were publications in which basic petroleum emollients [32,33], high lipid content emollients [40], and products with additional substances were used (such as probiotic lysate [36], ceramides [35], and shea butter) [15]. The emollient types used in the intervention groups differed and included formulas such as creams, oils, gels, balms, and emulsions. The systemic review and meta-analysis by Junqin Liang et al. [45] showed that emollient emulsion might be the superior option for preventing AD development in infants. Emollients may contain lipids that are nonphysiological, including petrolatum or lanolin, or physiological, which mimic lipids naturally present in the stratum corneum such as ceramides and cholesterol. The mechanism of action varies between the emollients due to their lipid content.

The side effects of emollient application include skin infections, which were reported in the BEEP trial [48]. Some authors also drew attention to the increased risk of infant slippage among emollient-treated groups [47].

The studies discussed above showed a lack of information about the risk factors for AD in the research group. The children in these studies were mostly divided into groups on the basis of the history of atopy in the family; however, other risk factors, including different climatic zones, urban versus rural settings, diet, breastfeeding and time of weaning, tobacco smoke exposure, and pollution [49], were ignored. The populations examined in the trials were heterogeneous, and the AD incidence varied in the different populations, which made it problematic to adequately compare the studies.

The diagnostic criteria for AD also differed in the discussed studies. Moreover, with a skin-product application, compliance is utterly important in the trials. Compliance in the PreventADALL trial was suboptimal, with full protocol adherence in only 32% of the participants in the skin intervention group. This observation should be considered when analyzing the effect of the intervention, but it may also prove that AD treatment could be inconvenient for the patients and their families.

The dosage of the emollients used in the mentioned studies varied: in several studies. Parents were obliged to apply emollients for at least 5 days per week in some studies, and in some others, the product was administered solely on the face (PreventADALL Study). Regular, daily emollient use in the intervention groups was observed to protect from AD. In contrast, studies that did not prove the effectiveness of emollients required the products to be used three-to-four times per week or only on the face (PreventADALL/BEEP trial). Repeated emollient application on normal skin twice a day for 1 week appeared to increase the hydration of stratum corneum and maintain it for more than 1 week after the end of treatment [50].

The treatment in the studies also started at various ages of the examined children, which made it difficult to compare their results.

In the STOP-AD randomized trial, the authors also measured gene encoding filaggrin status, which resulted in the conclusion that children with the FLG mutation may benefit more from early emollient intervention than FLG wild-type children.

Notably, emollients may contain haptens, which are small molecules that elicit an immune response when attached to a large carrier such as a protein. In their study, Kunkiel K et al. created a database of all products and compared their composition with 139 contact haptens listed in the European Baseline Series and the Fragrance and Cosmetic Series. The study showed that the vast majority of emollients contain at least one potential contact hapten [51].

Emollients may act as a protective agent in some cases, and their regular use can prevent AD development, but they will not eliminate the other risk factors of AD among children.

It should be emphasized that emollients are expensive, which can generate concerns about their cost-effectiveness in AD prevention. Tracey H. Sach et al. analyzed the cost-effectiveness of the BEEP trial and found that emollient treatment during the first year was not cost-effective for preventing AD [52]. Another cost-effectiveness analysis of emollients in preventing relapse among patients with AD showed that using emollients to prevent flares in AD is cost-effective [53]. In the mentioned study, five emollients were tested, and the application of the most effective emollient cost EUR 1575.64 for 5 years of treatment, and the effectiveness was 3.89 years without flare-up. The least effective emollient was emollient E, and the time without flare-up reached 3.80 years. In the study, the expenses also contained the cost of medical consultations, topical glucocorticosteroids, hospitalization costs, and follow-up costs of medical practitioners (i.e., generalists and specialists). The group without emollient use exhibited the highest cost of medical expenses in total. Generally, if emollient intervention was effective in AD prevention, it would be cost-effective, due to the reduced overall burden of medical expenses for AD treatment in the future, but the results are inconclusive.

## 5. Conclusions

AD is not just a health problem but also a social problem, as it leads to expenses, absenteeism from work and school, and avoidance of social interactions [1]. The median annual out-of-pocket expense for AD treatment in the United States (US) is USD 600 and may be USD 1000 or greater for over 40% of patients and families [54].

It seems that the strongest influences on AD development in high-risk children are genetics, in-utero programming, and immunology [11].

Is it possible, based on current knowledge, to support the preventive role of emollients in AD? Emollient treatment has a good safety profile, and most of the ingredients in emollient formulations are nonirritant for sensitive newborn and infant skin. There is some evidence of the positive effects of emollient application in preventing AD in predisposed populations. The relatively high cost of emollient treatment (vs a regular infant skin-care routine) would support the necessity for further evaluation of the effectiveness of emollients in nonpredisposed populations. The articles analyzed in our study showed that regular emollient usage can delay onset, decrease topical corticosteroid demand, and improve quality of life. Nevertheless, evidence of the effectiveness of emollients in AD prevention is ambiguous.

Emollients have different ingredients, and it is still unclear which composition might be of the greatest benefit in AD prophylaxis.

Considering the best interest of patients and their families, as well as the demands of society, there is a need for extensive research that will investigate all risk factors of AD and compare emollients with different ingredients.

## Figures and Tables

**Table 2:** Overview of meta-analysis of emollient usage for the prevention of atopic dermatitis in infants.

Article	Number of Articles and Participants	Population	Intervention	Relative Effect	Conclusion
Xu D et al. (2022) [42]	9 RCT Intervention = 1483 Control = 1509	0–12 months	Daily use of emollient vs. no regular administration	RR = 0.7 CI = 0.48–1.01	No statistically significant difference in incidence rate of AD
Priyadarshi M et al. (2022) [43]	2 RCT *N* = 1408 Intervention = 695 Control = 713	0–28 days on-term babies AD diagnosis up to 1 year No risk factors of AD	Emollient application vs. no emollient application	RR = 1.29 CI = 0.96–1.72	No difference in the incidence of AD at 12 months of age
Priyadarshi M et al. (2022) [43]	11 RCT *N* = 1988 Intervention = 1015 Control = 1022	0–28 days on-term babies AD diagnosis up to 1 year Risk factors of AD	Emollient appliance after bathing, at least four days a week vs.	RR = 0.74 CI = 0.55–1.00	Intervention probably lowers the risk of atopic dermatitis among ‘at risk’ newborns
Kelleher MM et al. (2022) [44]	33 RCT *N* = 25,827	0–14 days almost all participants	Skin barrier intervention versus standard care or no skin-care intervention	RR = 1.03 CI = 0.81–1.31	Skin care interventions such as emollients during the first year of life in healthy infants are probably not effective for preventing eczema, and probably increase risk of skin infection.
Liang J et al. (2023) [45]	11 RCT *N* = 3483 Intervention = 1740 Control = 1743	0–12 months	Early application of emollients vs. no treatment in high-risk infants	RR = 0.64 CI = 0.47–0.88	Early application of emollients is an effective strategy for preventing AD development in high-risk infants
Zhong Y et al. (2022) [46]	2 RCT *N* = 1349 Intervention = 713 Control = 716	0–6 weeks general population	prophylactic emollient treatment vs. placebo or no treatment	RR = 0.84 CI = 0.64–1.01	No significant reduction in the development of AD
Zhong Y et al. (2022) [46]	8 RCT *N* = 2158 Intervention = 1033 Control = 955	0–6 weeks high risk for AD, based on strong family history	prophylactic emollient treatment vs. placebo or no treatment	RR = 0.75 CI = 0.43–0.81	significant benefit of prophylactic emollients in the high-risk population
Kelleher MM et al. (2021) [47]	7 RCT *N*= 3075 Intervention = 1489 Control = 1586	0–12 months	Skin care intervention compared to standard skin-care or no skin-care intervention	RR = 1.03 CI= 0.81–1.31	Skincare interventions probably do not change risk of eczema but they probably increase risk of local skin infections, and may increase risk of infant slippage

RCT-randomized controlled trial.
